# Genetic diagnosis and treatment of a Chinese ketosis-prone MODY 3 family with depression

**DOI:** 10.1186/s13098-016-0198-5

**Published:** 2017-01-17

**Authors:** Jun Tang, Chen-Yi Tang, Fang Wang, Yue Guo, Hao-Neng Tang, Ci-La Zhou, Shu-Wen Tan, Shi-Ping Liu, Zhi-Guang Zhou, Hou-De Zhou

**Affiliations:** Department of Metabolism and Endocrinology, National Clinical Research Center for Metabolic Disease, The Second Xiang-Ya Hospital of Central South University, 139 Middle Ren-Min Road, Changsha, China

**Keywords:** MODY, HNF1A, Mental health, Ketosis-prone diabetes

## Abstract

**Background:**

To analyze the gene mutation and mental disorder of a Chinese ketosis-prone diabetes (KPD) family, and to make a precise diagnosis and give a treatment for them.

**Methods:**

We studied a Chinese family with a clinical diagnosis of maturity-onset diabetes of the young (MODY). The clinical data and the blood samples were collected. The promotor and coding regions inclusive intron exon boundaries of the HNF1A, HNF4A were detected by polymerase chain reaction (PCR) and direct sequencing. The missense mutation was also analyzed by bioinformatics. Genetic counseling was performed twice a month to relieve the mental disorder of the persons.

**Results:**

The missense mutation c.779 C>T (p.T260M) in exon4 of HNF1A gene was detected, and the symptom heterogenicity among persons in this family were found. All the members were retreated with Gliclazide and stopped to use other medicine, the blood glucose of them were well controlled. We also performed an active genetic counseling to them and the mental disorder of the proband’s sister was relieved.

**Conclusions:**

A missense mutation of HNF1A gene was first found in Chinese ketosis-prone MODY family with manifestations heterogenicity among the persons. Sulphonylureas medicine and genetic counseling are efficiency ways to treat MODY 3 and its’ mental disorder respectively.

## Background

Monogenic diabetes (MD) is genetically and clinically heterogeneous, which includes maturity-onset diabetes of the young (MODY), infancy-onset and neonatal diabetes mellitus and many rare forms of atypical diabetes, which accounts for approximately 1–2% of all diabetes, and is difficult to distinguish from type 1 and type 2 diabetes mellitus [1, 2]. MODY is reported to be the most common form of MD and there are 14 MODY subtypes have been listed on the Online Mendelian Inheritance in Man (OMIM) database [3, 4]. Mutations in the GCK, HNF1A, HNF4A, and HNF1B genes are the most common causes of MODY in the UK, and they represent different clinical characteristics respectively. The MODY2 persons got mild, subclinical hyperglycemia, which is generally present at birth and does not progress. MODY 1 and MODY 3 persons are tendency for microvascular complications and sensitivity to sulfonylureas [3]. MODY 3 is caused by hepatocyte nuclear factor-1A (HNF1A) gene mutation (also named as HNF1A-MODY), which accounts for 30–50% of MODY [3]. However, the mutations of HNF1A gene were detected in only 9% Chinese people with MODY, and a majority of Chinese MODY people are due to the defection of unknown genes [5]. As absolute insulin deficiency is not typical for HNF1A-MODY persons, the development of diabetic ketoacidosis (DKA) in a person with HNF1A-MODY is rare and just two previous cases are described [6, 7], it is estimated that approximately 80% of MODY are misdiagnosed as type 1 or type 2 diabetes mellitus [8]. The limitations in physician’s awareness and the restrictions in performing genetic testing are common reasons [9]. There are lots of studies focused on the relation between the young people with diabetes and mental health, no matter people with type 1 or type 2 diabetes mellitus, both of them are needed more psychological support [10]. Here, we reported a missense mutation of HNF1A gene was first found in Chinese ketosis-prone MODY family with manifestations heterogenicity among the persons. Sulphonylureas medicine and positive genetic counseling are efficiency to treat MODY 3 and its’ mental disorder respectively.

## Methods

### Clinical studies

The study was approved by the Ethics Committee of the Second Xiang-Ya hospital of Central South University. Informed consent was obtained from all participants. Clinical information was obtained, which contain the current history, family history and so on.

### Genetic analysis

Genomic DNA was extracted from blood samples with commercial kits (Qiagen). The previous descripted primers were used for exons, the intron–exon boundaries and the promoter regions of HNF1A and HNF4A were screened for mutations by polymerase chain reaction (PCR) amplification, and direct sequencing of the PCR products was performed as described previously [11].

### Islet autoantibody assay

Serum antibodies to GAD, IA2, and ZnT8 were measured by radio binding assay with in vitro translated 35S-methionine-labelled GAD65, IA- 2, or ZnT8 [12].

### Islet autoantibody assay

The participants were diagnosed as MODY3 and estimated the risk to develop the disorder to their relatives and/or to transmit it to offspring. Talking with the participants and their family twice a month in the first three months, and a systemic education was given to the patients to relieve the mental stress.

## Results

**Fig. 1:**
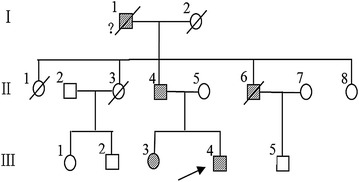
Pedigree of the family. The *arrow* indicates the proband. *Squares* represent males and *circles* females, diabetic persons are indicated with *filled symbols*. The proband (III4) was born in 1993, and diagnosed with diabetes in 2014. His sister (III3) was born in 1987, diagnosed with diabetes with no classification in 2005, and diagnosed with type 1 diabetes mellitus in 2008. The proband’s father (II4) was diagnosed with diabetes in 2009 when he was 50 years old. The proband’s uncle (II6) was diagnosed with diabetes in 2000 and died 5 years later. The proband’s grandfather (I1) was doubted suffered from diabetes but without medical records to make sure

The proband (III4 Fig. 1) was born in 1993 and diagnosed with diabetes in 2014. He was admitted to our hospital because of polydipsia, polyuria and weight loss for 6 months. His BMI was 19.96 kg/m^2^, fasting blood glucose was 8.32 mmol/L, 2 h-postprandial glucose was 20.02 mmol/L, glycated hemoglobin (HbA1c) was 54 mmol/mol (7.1%), fasting c-peptide was 341.3 pmol/L, and 2-h postprandial C-peptide was 926.4 pmol/L. The islet autoantibodies glutamate decarboxylase (GAD), islet antigen-2 (IA-2) and zinc transporter 8 (ZnT8) were negative. The high-sensitivity C-reactive protein (hs-CRP) was 0.09 mg/L. Then, he was treated with gliclazide and metformin to control the blood glucose. The clinical features of the four participants are shown in Table 1.

**Table 1:** Clinical features of patients of the pedigree with maturity-onset diabetes of the young (HNF1A-MODY)

	III3	III4	II4
Age (years)	27	22	55
Age at diagnosis (years)	18	21	49
BMI (kg/m^2^)	16.45	19.96	18.90
HbA1C mmol/mol (%)	–	54 (7.1)	–
FBG (mmol/l)	6.09	8.32	3.34
2-h PPG (mmol/l)	11.65	20.02	–
Fasting C-peptide (pmol/L)	114.9	341.3	–
2-h postprandial C-peptide (pmol/L)	–	926.4	153.7
GAD-Ab	Negative	Negative	Negative
IA2-Ab	Negative	Negative	Negative
ZnT8-Ab	Negative	Negative	Negative
Creatinine (μmol/L)	59.9	69.0	66.3
Urea nitrogen (mmol/L)	5.23	–	4.16
Uric acid (μmol/L)	315.2	280.5	260.4
Alanine aminotransferase (U/L)	11.4	11.9	23.9
Aspartate aminotransferase (U/L)	23.1	15.8	33.4
Triglycerides (mmol/L)	1.06	1.24	0.87
Total cholesterol (mmol/L)	5.49	4.98	4.02
HDL-C (mmol/L)	1.57	–	1.41
LDL-C (mmol/L)	3.53	–	2.19
Hs-CRP (mg/L)	0.09	0.28	–
Medication	Insulin	Sulphonylurea	Sulphonylurea

The proband’s sister (III3) was born in 1987 and diagnosed with diabetes in 2005. She was admitted to local hospital because of typical diabetic symptoms, and then was treated with glipizide, resulted in well blood glucose control and without hypoglycemic episodes, until to 2008. She was admitted to our hospital because of stopping taking glipizide for 3 months and with dizziness, vomiting and abdominal pain. Her fasting c-peptide was 201.65 pmol/L, fasting blood glucose was 11.8 mmol/L and the urine ketones were 100 mg/dL. The hs-CRP was 0.28 mg/L. The diabetic ketosis was defined as urine ketones ≥80 mg/dL in the absence of acidosis [13]. Then she was diagnosed as ketosis-prone type 1 diabetes mellitus (KPD), the treatment was switched to the insulin aspart 30 and later a long-acting insulin analogue (glagine), and she got a well blood glucose control. The C-peptide level records of the person III3 are show in Table 2.

**Table 2:** The C-peptide level records of the patient III3

Date	Fasting C-peptide	2-h postprandial C-peptide	Reference value
2008.7.31	201.65	–	223.40–746.20 pmol/l
2009.4.6	205.84	284.62	
2009.9.23	230.41	459.65	
2012.2.20	0.660	1.790	0.81–3.85 ng/ml
2012.11.11	0.610	1.260	
2013.7.22	0.350	2.480	
2015.9.27	0.680	1.570	

The proband’s father (II4) was born in 1959 and diagnosed with diabetes in 2009, he had not any clinical symptom related to diabetes before 2009. He tested his blood glucose because of the mild polydipsia, polyuria for almost 1 year, and the blood glucose was mild increased. He took the metformin to control the blood glucose. In 2013, he switched his treatment from metformin to glipizide by himself. From 2013 to 2014, he got sweating, palpitation, hunger for several times but no blood glucose data could be available when he got these symptoms. These symptoms indicated that he suffered from hypoglycemia.

**Fig. 2:**
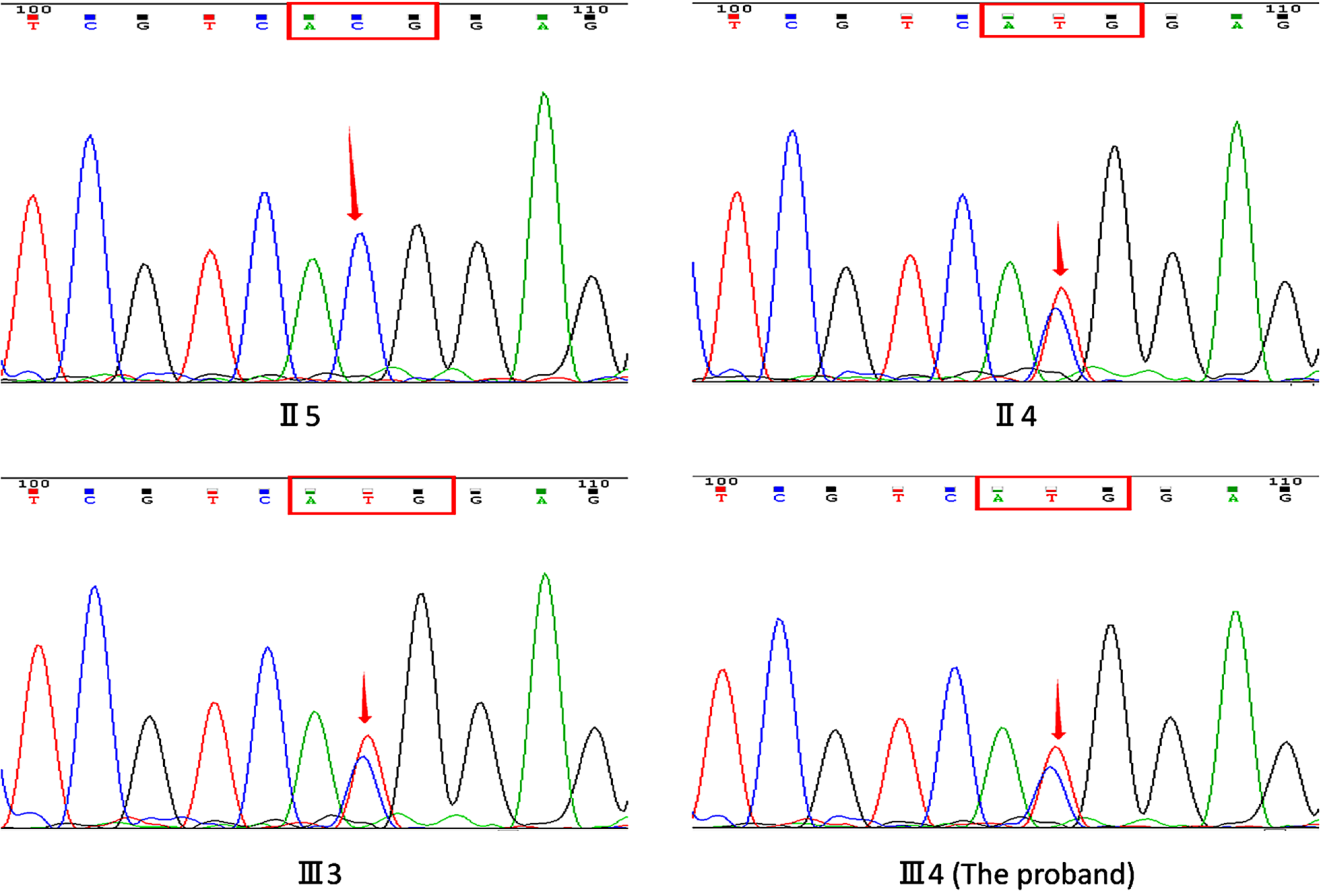
Sequencing chromatograms of the family members (*red arrow* represents the mutation site). The genetic testing results of the family. A missense mutation c.779 C>T in exon 4 of HNF1A gene was detected in the proband (*III4*), his sister (*III3*), and his father (*II4*). No mutation was found in the same gene of his mother. This mutation was reported as a pathogenic gene of HNF1A-MODY

According to the diagnosis age, insulin non-dependent at the onset and three consecutive generations were affected with obvious autosomal dominant inheritance. We considered the diagnosis was MODY. Because of the drug history and hypoglycemia symptoms of the proband’s father, they were suspected to have HNF-1A or HNF-4A mutation. All people were given written informed consent before the genetic testing. As a result, the direct sequencing showed a heterozygous missense mutation c.779C>T (p.T260M) in exon4 (Fig. 2), which was reported strongly associated with HNF1A-MODY. Two polymorphisms c.1375C>T (p.L459L), c.1460G>A (p.S487N) in exon7 of HNF-1A gene were detected, too.

**Fig. 3:**
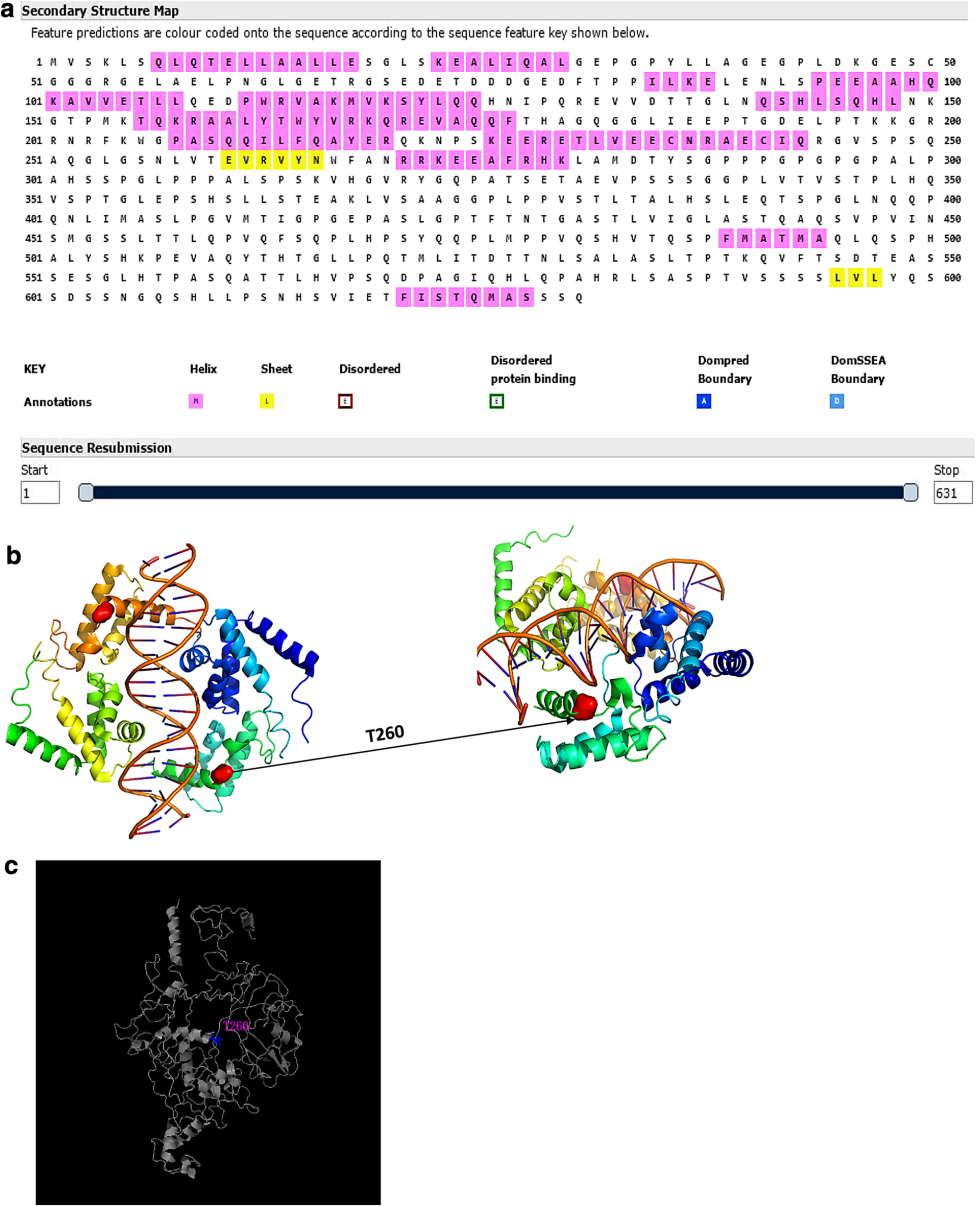
The protein structure and function predictions. **a** Analysis of MODY 3 secondary structure. **b** Highlight of point mutation T260 at the 3D structure of MODY3. **c** The T260M mutation analysis, the STRUM results showed the T260M ddG is −0.15

According to the gene sequence, we estimated the secondary structure of T260M mutated HNF1A using software PSIPRED (Fig. 3). The position 260 is located near to a sheet structure. We thereafter performed protein 3D structure simulation using I-TASSER. Full-length amino acid sequence of HNF-1A (reference strain: NG_011731.2) was imported into I-TASSER and from the COACH result, the T260 is near nucleic acid binding site [14–17]. The STRUM result (Fig. 3) showed that the mutation destabilize the protein [18].

Then, their treatments were switched. The proband was treated with gliclazide MR 30 mg per day, his sister switched insulin to glimepiride 2 mg per day, and the dosage of gliclazide was decreased to his father. The proband got insomnia, anorexia, fatigue and lost interest for almost everything and then he was admitted to the psychiatric clinics. Because he worried about the diabetes would deteriorate soon and then he would die like his uncle in that young age; and he also worried about the inheritance of the diabetes, that he even could not found a family. The psychiatrist made a diagnosis of depression and anxiety state. He was treated with cymbalta and estazolam. After the gene diagnosis, the proband was obtained with the positive genetic counseling, which provided him the clinical health care, education, and especially emotional support to face this disease. Finally, the person was recovered and do not need antidepressant medication anymore.

## Discussion

In this study, we reported a HNF1A-MODY family with a missense mutation in the exon 4 of HNF-1A gene c.779 C>T (p.T260M) which was first reported in 1997 [19], but it was the first time to be detected in Chinese population.

To date, although there were 414 different mutations of HNF1A-MODY have been reported worldwide [20], seldom of them were reported in China. Misdiagnosis were one of the reasons. The proband’s sister was diagnosed with type 1 diabetes mellitus at age 21. Her C-peptide could be detected (Table 2) and islet autoantibodies were negative, and her ketonuria were positive and without family history because the proband and his father had not diagnosed with diabetes at that time. In order to prevent the development of ketoacidosis, the insulin was taken to control the blood glucose and improve the metabolism. Even though there were two cases reported three people with HNF1A-MODY developed to severe DKA [6, 7], the present diagnostic recommendations suggest that the people with DKA would be excluded from MODY [21]. The proband’s sister was not developed to DKA in this study probably because of the timely treatment, but the ketosis-prone clinical situation led to the misdiagnosis. According to the study of UK there were 5% of the people who were diagnosed with diabetes before the age of 45 suffering from MODY, but 80% of them misdiagnosed with type 1 or type 2 diabetes mellitus, which suggested that without considering the family history of diabetes or metabolic features, all people who were diagnosed up to the age of 30 with diabetes and C-peptide ≥0.2 nmol/L should be considered for HNF1A and HNF4A genes test [8]. If the proband’s sister received the gene test at her first visiting, she will exempt from the insulin treatment.

The clinical phenotypes of the HNF1A-MODY people were heterogeneous even in a family with the same mutation, and both the environment and genetic factors may contributed to this phenomenon [22, 23]. Harries et al. discovered that the position of the mutation in the HFN1A gene was related to the age at the diagnosis of diabetes, and the age was lower if the missense mutations affecting the dimerization/DNA-binding domains than those affecting the transactivation domain. The 3D protein structure prediction has shown that the T260 was in the DNA-binding domains and the mutation T260M destabilize the protein, which indicates that the function of the protein has been affected and induced the diabetes. By Cotransfection experiments, Ekholm et al. showed that p.T260M mutation reduced the induction of the farnesoid X receptor (FXR) promoter and reduced the induction of the apical sodium-dependent bile salt transporter promoter [24], which indicated that p.T260M mutation leads to the functional changes of related gene. Three HNF1A isoforms were affected when the mutations were located in exon 1–6, and the diagnosis age of diabetes were younger than those with missense mutations involving one or two isoforms [25]. The missense mutation c.779 C>T was in exon 4 of this family, and that why the proband and his sister were diagnosed with diabetes at 21 and 18 years respectively. But their father got the diabetic symptoms and diagnosed at 50 years. The different lifestyles of two generations, and the proband’s father, as a farmer, should always do farm work in his daily life, may play a role to delay the age at onset of diabetes effectively.

Sulfonylureas have been demonstrated could treated individuals with HNF1A-MODY effectively by acting on ATP-sensitive potassium channels [9]. Shepherd et al. discovered that the majority of people with MODY3 could transferred insulin to sulfonylureas successfully without deterioration in glycemic control [26]. The proband’s older sister, who had taken the insulin to control blood glucose for almost 7 years, switched her treatment to glimepiride. The proband was treated with gliclazide only. The dosage of gliclazide was decreased for their father to avoid hypoglycemia episode. All the blood glucose of them was improved after changing the treatment (For the proband’s sister the HbA1c decreased from 58 to 52 mmol/mol (7.5–6.9%). The treatment of proband’s father played a key role in further gene test, which narrowed the gene screening scope. The medication history of clinical diagnosed MODY persons should be paid more attention to help doctors choose the appropriate gene test.

Mental disorder, such as depression and anxiety, in diabetes people have been aroused a lot of attention in recent years. Compared to the people without diabetes, the prevalence rate of depression is more than three-times higher in people with type 1 diabetes mellitus and nearly twice as high in people with type 2 diabetes mellitus [27]. Browne et al. founded that the young adults with type 2 diabetes mellitus were more likely to have clinically meaningful depressive symptoms than the older adults [10]. They thought compared with the older type 2 diabetes mellitus people, the young adults with type 2 diabetes mellitus may need more intensive psychological and self-care support [10]. The mental disorder such as depressed mood may be lead to poor glycemic control [28]. The proband got metal disorder because several of his families got diabetes, and he thought the disease may be inherited to next generation. The possibility of insulin use may be another reason induced the mental disorder of the proband. After the diagnosis of MODY3 and the positive genetic counseling, the proband knew that this type of MODY is positively to sulphonylureas and he can have healthy children in future with the development of the genetic medicine. The timely genetic counseling is crucial to relief depression symptom of the proband and he doesn’t need the anti-mental disorder medications.

The timely and positive genetic counseling is an effective way to help persons and their family to make informed, autonomous health care and reproductive decisions by providing the clinical health care, education, and emotional support [29, 30], which will relief their mental pressure.

## Conclusions

In summary, a missense mutation of HNF1A gene was first found in Chinese ketosis-prone MODY family with various clinical manifestations in different members. Sulphonylureas medicine and genetic counseling are efficiency ways to treat MODY 3 and its’ mental disorder respectively.

## Abbreviations

MODY: maturity-onset diabetes of the young
HNF1A: hepatocyte nuclear factor-1A
GAD-Ab: glutamate decarboxylase antibody
IA2-Ab: protein tyrosine phosphatase antibody
ZnT8-Ab: zinc transporter 8 antibody
HbA1c: glycated hemoglobin
hs-CRP: high-sensitivity C-reactive protein
BMI: body mass index
FBG: fasting blood glucose
PPG: postchallenge plasma glucose
